# Country-level heterogeneity in MDR-TB drug susceptibility supports country-specific policy development

**DOI:** 10.3389/ftubr.2025.1667354

**Published:** 2025-09-25

**Authors:** Naomi M. Fuller, Nicholas G. Davies, Timothy D. McHugh, Gwenan M. Knight

**Affiliations:** ^1^Department of Infectious Disease Epidemiology, Faculty of Epidemiology and Population Health, London School of Hygiene and Tropical Medicine, London, United Kingdom; ^2^Centre for Mathematical Modelling of Infectious Diseases, London School of Hygiene and Tropical Medicine, London, United Kingdom; ^3^Antimicrobial Resistance Centre, London School of Hygiene and Tropical Medicine, London, United Kingdom; ^4^Tuberculosis Centre, London School of Hygiene and Tropical Medicine, London, United Kingdom; ^5^Centre for Clinical Microbiology, Division of Infection & Immunity, University College London, London, United Kingdom

**Keywords:** bedaquiline drug susceptibility, multidrug-resistant tuberculosis, phenotypic drug resistance, minimum inhibitory concentrations, country variability, MIC distribution analysis, resistance surveillance data

## Abstract

**Objectives:**

The global challenge of tuberculosis (TB) is exacerbated by multidrug-resistant TB (MDR/RR-TB), confounded by country-level differences in TB prevention and care. The impact of local differences in drug-resistance selection pressure and transmission may be observed by analyzing distributions of minimum inhibitory concentrations (MICs). Using the Bedaquiline Drug-Resistance Emergence Assessment in MDR-TB (DREAM) dataset, we analyzed MIC distributions derived from a standard protocol across 11 countries and 12 antibiotics to explore country-level variation in drug susceptibility.

**Methods:**

We analyzed 71,135 MICs from 5,928 MDR/RR-TB isolates sampled from bedaquiline-naive patients. We compared MIC distributions across countries and WHO resistance classes, then used Spearman rank correlations to compare the drug-susceptibility within individual isolates by country. To explore the effect of bedaquiline use on resistance, we used linear regression to compare bedaquiline MICs with WHO data on bedaquiline usage.

**Results:**

MIC distributions between countries were heterogeneous, especially for fluoroquinolones and isoniazid. The correlation analysis revealed a relationship between bedaquiline and clofazimine MICs in six countries. Analysis of isolates by resistance class demonstrated that XDR-TB isolates had higher MICs than MDR-TB isolates for antibiotics not part of the XDR definition. We found limited evidence to suggest that past bedaquiline usage at the national level led to raised bedaquiline MICs in patients not exposed to the drug.

**Conclusions:**

Our research shows clear variations in drug susceptibility within *M. tuberculosis* across different countries and resistance classes, providing evidence of distinct drug-susceptibility dynamics per country. This expands the evidence for MDR-TB country differences and supports further country-specific policy development.

## Introduction

Antibiotic-resistant strains significantly exacerbate the ongoing global challenge of tuberculosis (TB). In 2023, the estimated incidence of resistance to the key first-line drugs isoniazid and rifampicin—i.e., multidrug-resistant- and/or rifampicin-resistant TB (MDR/RR-TB)—reached 400,000 cases (1). Regularly updated international guidelines for TB treatment and diagnosis are outlined by the World Health Organization (WHO) (2). However, marked disparities exist in TB prevention and care across different countries (1, 3–5). It is imperative to understand how these country-level policy variations may drive the selection, evolution, and transmission of drug resistance in TB globally and locally to formulate effective strategies to end TB.

The burden of MDR/RR-TB is uneven: in 2023, 42% of the estimated incidence of MDR/RR-TB cases were concentrated in three countries (India, Philippines and Russia) (1). Variations in drug-susceptibility may reflect differences in local epidemiological conditions, transmission dynamics, treatment practices, and resistance mechanisms. WHO regularly produces country-specific TB reports quantifying how indicators of TB epidemiology differ geographically, such as the rate of change in MDR/RR-TB incidence (6). For example, Pakistan's TB programme investments have increased MDR/RR-TB notifications and the number of patients receiving treatment (7). South Africa has shown a marked decrease in MDR/RR-TB notifications because of the disruption caused by the COVID-19 pandemic (6). To bring attention to specific high-burden countries, WHO has categorized countries into three lists since 2016: high TB burden, high TB/HIV burden and high MDR/RR-TB burden (8).

WHO's drug-resistant TB (DR-TB) treatment guidance often undergoes policy revisions, with one of the more significant changes being the definition of extensively drug-resistant TB (XDR-TB) (5). Initially defined in 2006 as MDR-TB with additional fluoroquinolone (specifically, levofloxacin or moxifloxacin) resistance and resistance to an injectable second-line drug (capreomycin, kanamycin or amikacin), in 2021, the category was subdivided into pre-XDR (MDR/RR-TB plus levofloxacin or moxifloxacin resistance) and XDR-TB (pre-XDR-TB plus bedaquiline and/or linezolid resistance) (9). Treatment recommendations have also evolved substantially, progressing from the introduction of bedaquiline-containing MDR-TB treatment in 2013 to the establishment of the shortened bedaquiline, pretomanid, linezolid, and/or moxifloxacin (BPaL/M) regimen by 2022 (2, 10–12). As these evolving treatment guidelines will be implemented at different rates in different settings, there may be substantial differences in bacterial evolution and transmission dynamics across different geographical contexts. South Africa has reported an increased prevalence of bedaquiline resistance following several years of use of the drug (13). Understanding such geographical variations in drug-susceptibility is crucial for predicting BPaL/M's long-term effectiveness and informing policy decisions to prevent resistance spread. This consideration becomes particularly important given global inequities in antibiotic access and quality control, which can lead to varying treatment outcomes across different countries (14–17).

Minimum inhibitory concentration (MIC) testing determines drug susceptibility by identifying the lowest antimicrobial concentration that prevents detectable bacterial growth *in vitro* (18). In bacterial populations, such as *Mycobacterium tuberculosis*, MICs reveal the spectrum of resistance levels, with concentrations below an epidemiological cut-off considered unlikely to affect treatment success but which may give insights into MIC changes over time (“creep”) and future treatment failure. This variation stems from genotypic (resistance-conferring mutations) and phenotypic (e.g., tolerance) mechanisms (19). Previous studies of other bacteria have shown that MIC distributions can vary significantly between patient demographics, such as age groups and sex (20). We, therefore, hypothesize that *M. tuberculosis* MIC distributions will reflect geographical variations and reveal bacterial heterogeneity across populations. When comparing across resistance definitions, we expect MICs to remain stable for antibiotics that are common between definitions, with changes only observed for newly included antibiotics. For example, XDR isolates should have similar rifampicin MICs to MDR isolates (rifampicin resistance is in both definitions) but show elevated MICs for bedaquiline (unique to XDR-TB definition). MIC variation offers insight into the evolutionary dynamics of *M. tuberculosis*, extending beyond its role in defining ECOFFs (21). Shifts in MIC values, particularly above the ECOFF, highlight traits that may confer a selective advantage under drug pressure (22). Through this analysis of MIC distributions, we aim to gain insights into the evolutionary patterns of drug susceptibility in *M. tuberculosis*.

We used the Bedaquiline Drug-Resistance Emergence Assessment in MDR-TB (DREAM) dataset of MICs measured across 11 countries to explore differences in drug susceptibility per country (23). The MICs in the DREAM dataset were obtained through isolate testing using the same protocol and assay implemented across different laboratories in each country. This reduces variation in results based on laboratory protocol, allowing for a comparison of country-level variation. The study population consisted of bedaquiline-naïve TB patients, meaning any bedaquiline resistance identified did not emerge due to drug selection. Previous analysis showed differences in bedaquiline resistance prevalence between countries (3.4% of isolates in South Africa vs. 0% in Thailand) and by resistance class (0.9% of XDR-TB isolates (2006 definition) vs. 0.3% of MDR-TB isolates) (23). However, no country-level differences among MICs for other antibiotic classes were examined.

This paper explores country-level variations in drug susceptibility to support policymakers in developing country-specific interventions and treatment designs to end TB, building on the initial DREAM analysis and methodology for exploring MIC distributions (20, 23). A particular focus was placed on understanding the implications for TB treatment regimens, particularly bedaquiline, which is challenging to detect resistance to (24). We hypothesized that various country-specific factors influence the evolution of drug resistance in *M. tuberculosis* as observed in MIC distributions. To investigate this hypothesis, we established four key objectives: first, to characterize the MIC distribution differences between countries; second, to explore correlations in MIC levels within isolates per country to potentially predict unknown MICs; third, to analyse isolate MIC differences within resistance classifications; and fourth, to evaluate the relationship between country-isolate MIC differences and past bedaquiline use.

## Methods

### Data cleaning and preparation

Access to the DREAM dataset was acquired through the Vivli AMR Register (25, 26). This contained data from 2011 to 2019 and 5,928 isolates. Data cleaning and part of the analysis were adapted from a similar non-TB project (20). We included the following information from the DREAM dataset in our analysis: country (India, Lithuania, Pakistan, Philippines, South Africa, South Korea, Taiwan, Thailand, Turkey, Vietnam, and the United States), year of isolate collection, and MICs using 7H9 growth media for 12 antibiotics: isoniazid, rifampicin, linezolid, clofazimine, levofloxacin, ofloxacin, moxifloxacin, capreomycin, kanamycin, amikacin, ethambutol and bedaquiline. Out of 71,135 MICs, 12,383 (17%) had a qualifier (> or ≤ ) for the upper and lower bounds only, where ≤ sign indicated the MIC was at the lower end of the range (e.g., < 1 was reported as ≤ 1), and > sign indicated the MIC was above the range (e.g., >1 was reported as ≥2). We applied the same logic but removed the qualifier (e.g., >1 to 2; ≤ 1 to 1). Only one MIC for capreomycin in a US isolate was missing from the data. We included the epidemiological cut-off (ECOFF) defined by Kaniga et al. for antibiotics other than bedaquiline and EUCAST for bedaquiline (Supplementary Table S1) (23, 27). We then assigned each country an income group from the World Bank, WHO region and WHO TB-priority group (Supplementary Table S2) (1, 28, 29). While resistance classifications were previously recorded in the database, we updated these to align with current WHO definitions, enabling a more contemporary evaluation (1). All data cleaning and analyses were conducted in R 4.4.2 (30).

### Characterizing country MIC differences

#### Between countries

Histograms of MIC distributions per country were plotted to enable comparison between countries. Data from all countries was presented under a “global” category. We also generated a histogram plot for MIC values per year. Cumulative plots of the number of isolates from each country, year, income group, WHO region, and WHO TB priority group per MIC were generated as in Wildfire et al. (20) to examine if MICs were heterogeneous between groupings. For each antibiotic, and within each country and year, we calculated the first quartile (Q1), second quartile (Q2), third quartile (Q3), and interquartile range (IQR) of MIC values to characterize the distribution and central spread of resistance profiles.

#### Within countries

We conducted a Spearman rank correlation on MICs from single isolates for all antibiotic pairs at the level of individual countries and the global dataset. We then ranked the Spearman rank correlation coefficient ρ as negligible (|ρ| < 0.1), weak (0.1 ≤ |ρ| < 0.4), moderate (0.4 ≤ |ρ| < 0.7) or strong (|ρ| ≥ 0.7) according to standard practices (31).

### Characterizing bacterial MIC differences

Histograms and cumulative plots of MIC distributions per resistance classification were plotted, as well as calculating the quartiles and IQR. Several isolates were found to have a rifampicin MIC below the epidemiological cut-off; we defined these as “rifampicin-susceptible.” We treated isolates with a rifampicin MIC above the epidemiological cut-off as MDR/RR-TB isolates.

### Bedaquiline treatment implications

Bedaquiline usage data were obtained from the Global TB Programme from WHO reports on “TB Notifications” (32), encompassing the years corresponding with bedaquiline MICs in the DREAM database (2014–2019). We calculated the proportion of MDR/XDR-TB patients who initiated bedaquiline therapy as a proportion of all who initiated treatment per year (“mdrxdr_bdq_tx”/“conf_rrmdr_tx”) (33). The mean MIC value per year for the isolates was plotted against the proportion of MDR/XDR patients starting bedaquiline-containing therapy in the same year. A linear regression analysis was used using the “lm” function in the R programming language.

## Results

### Characterizing country MIC differences

**Figure 1:**
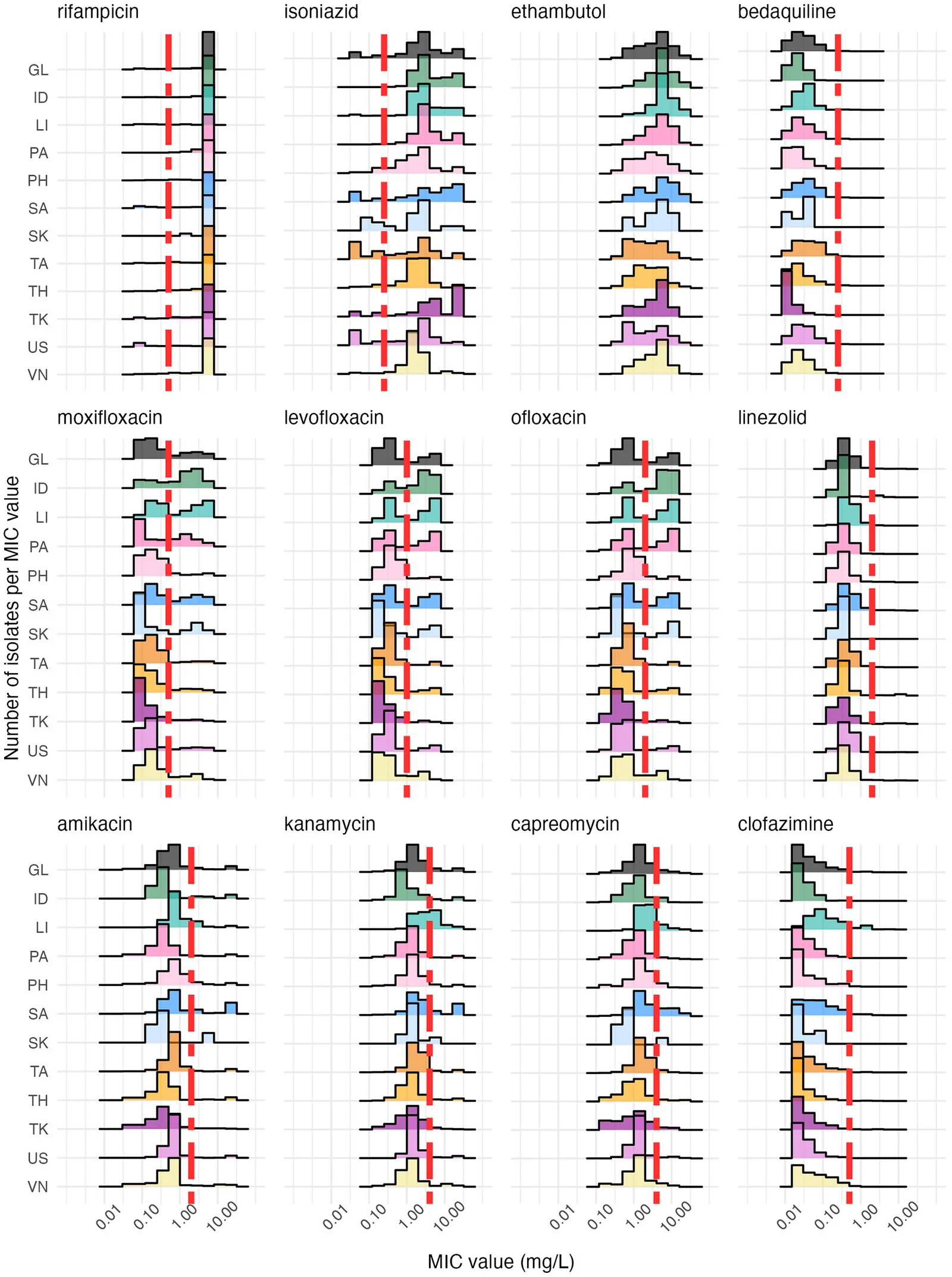
A histogram of MICs stratified by country (color) shows that each country has a distinctive antibiotic susceptibility profile, as indicated by the distribution of isolates (y-axis, number of values) over MIC values (x-axis, log scale). The vertical red dashed line is the epidemiological cut-off for each antibiotic, except for ethambutol, which has no reported epidemiological cut-off (Supplementary Table S1). The antibiotics are ordered by family, with the fluoroquinolones (levofloxacin, moxifloxacin, ofloxacin) in the middle row and the aminoglycosides (amikacin, capreomycin, kanamycin) appearing on the bottom row. The country codes along the y-axis are as follows: GL, Global; ID, India; LI, Lithuania; PA, Pakistan; PH, the Philippines; SA, South Africa; SK, South Korea; TA, Taiwan, TH, Thailand; TK, Turkey; US, United States; VN, Vietnam. The number of isolates per country can be found in Supplementary Table S3 and the percentage of resistance per country and antibiotic in Supplementary Table S4. Not every country reported isolates for all MIC levels for each antibiotic.

Our examination reveals heterogeneity in MIC distributions by country (Figure 1). MIC distributions for fluoroquinolones (levofloxacin, moxifloxacin, ofloxacin) show the greatest heterogeneity (e.g., range of IQRs for OFX 0.375–15.0 mg/L, Supplementary Table S5), suggesting considerable variation in use in different countries. For isoniazid, most heterogeneity (range of IQRs 2.0–28.0 mg/L, Supplementary Table S5) is seen above the epidemiological cut-off, often with two peaks, representing the multiple genetic mutations that can cause isoniazid resistance (34, 35). In contrast, most heterogeneity seen for bedaquiline (range of IQRs 0.01–0.09 mg/L, Supplementary Table S5) is below the epidemiological cutoff. Despite initial screening of DREAM isolates, some rifampicin-susceptible isolates are present.

When analyzing the distribution of bedaquiline MICs, South Africa emerges as an outlier with increased MICs (max MIC of 2.00 mg/L vs. Q3 = 0.06 mg/L, Supplementary Table S5), whereas Turkey is an outlier with significantly lower MICs (max MIC of 0.12 mg/L vs. Q3 = 0.015 mg/L, Figure 1, Supplementary Table S5). Taiwan has the highest Q3 value for bedaquiline of 0.12 mg/L (max MIC of 0.5 mg/L, Figure 1, Supplementary Table S5), indicating that its MIC distribution for bedaquiline is elevated toward the ECOFF compared to other countries, while also demonstrating minimal outliers. India, Pakistan and Lithuania have high MIC distributions for fluoroquinolones (OFX: Q3 = max MIC = 16 mg/L; LVF: Q3 = max MIC = 16 mg/L; MOX: Q3 = 4 mg/L & max MIC = 8 mg/L, Figure 1, Supplementary Table S5), closely followed by South Africa (LVF: Q3 = 4 mg/L, max MIC = 8 mg/L; MOX: Q3 = 3.5 mg/L, max MIC = 8 mg/L, Figure 1, Supplementary Table S5). South Africa consistently had elevated MIC distributions across antibiotics compared to other countries, with amikacin being a significant concern (Q3 = 4 mg/L, Supplementary Table S5). Conversely, countries like Turkey and the US demonstrated distributions skewed toward lower MIC values. These observations underscore the heterogeneous nature of DR-TB profiles globally. The global trend is an average and obscures the nuanced differences among countries for many antibiotics tested here.

Longitudinal analysis of MIC distributions by study year reveals an oscillating pattern that suggests an overall increase in MIC over time for the fluoroquinolones (e.g., IQRs for OFX from 2013 to 2019: 0.0, 0.5, 7.5, 3.5, 7.5, 3.0, 7.0 mg/L). A reduction in MIC values by study year for isoniazid was found (range of IQRs 2.0–14.0 mg/L) but no consistent changes over time for the remaining antibiotics (Supplementary Figure S1, Supplementary Table S5). Whilst heterogeneity between countries is evident in cumulative graphs, lower levels of heterogeneity can be seen when grouping by country-level income groups, WHO regions and TB-priority groups (Supplementary Figures S2, S3).

**Figure 2:**
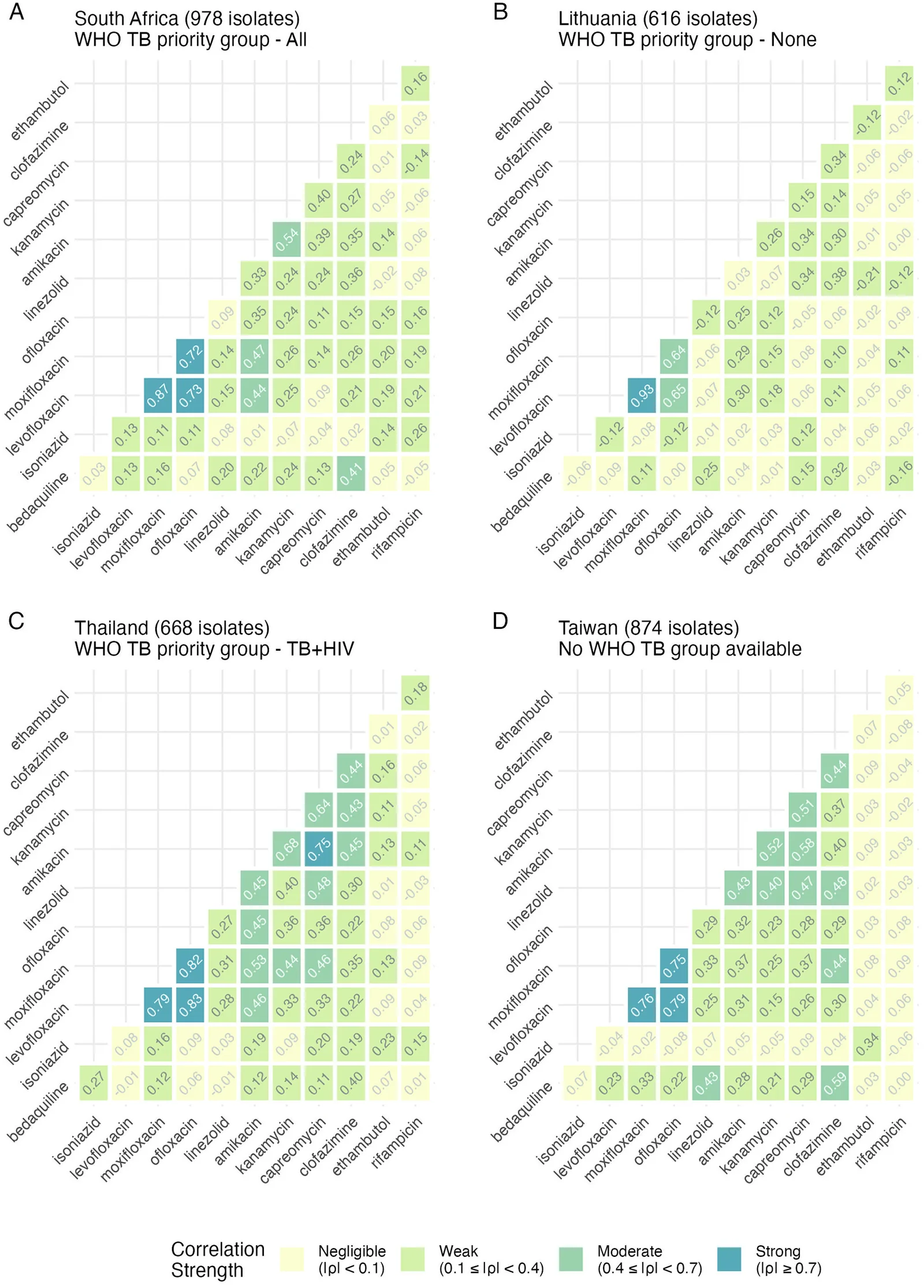
Spearman rank correlation analysis of antibiotic pairs within isolates by country reveals distinct relationships. Each panel **(A–D)** represents a country, with each tile containing the correlation result between each antibiotic shown on the axes. A higher value and a darker color represent a higher correlation. All correlations are colored along the same scale for easy comparison. Four example countries are shown here (South Africa, Lithuania, Thailand, and Taiwan), and the number of isolates per country is shown in the title. Where it exists, the WHO TB priority group is also indicated. Results for other countries are shown in Supplementary Figures S4, S5.

Correlation matrices of antibiotic MICs within isolates show distinct country-specific correlation profiles (Figure 2). The majority of correlations shown here are positive, though three moderate negative correlations were present in South Korea for clofazimine-ethambutol, capreomycin-ethambutol, and isoniazid-clofazimine (Supplementary Figure S4, Supplementary Table S6), with remaining negative correlations being negligible or weak (−0.4 < ρ ≤ 0). For all countries, there was a moderate (0.4 < ρ < 0.7) to strong (ρ>0.70) positive correlation within the antibiotic family of fluoroquinolones (levofloxacin, moxifloxacin, ofloxacin). The family of aminoglycosides (amikacin, capreomycin, kanamycin) have strong correlations in India, South Korea and Thailand (ρ ≥ 0.7; Supplementary Table S6), moderate correlations (0.4 < ρ < 0.7) in all other countries and weak correlations (0.1 ≤ ρ < 0.4) in Lithuania, South Africa and the US. Bedaquiline and clofazimine emerged as having moderate correlations (0.40 < ρ < 0.7) in South Africa, Taiwan, Pakistan, South Korea and the US. A moderate bedaquiline-linezolid correlation (ρ = 0.43) was present in Taiwan, and a moderate bedaquiline-kanamycin correlation was present in Turkey (ρ = 0.44). A moderate aminoglycoside-clofazimine correlation (0.40 < ρ < 0.7) was present in six countries: Pakistan, Philippines, Taiwan, Thailand, Turkey and Vietnam. Clofazimine-moxifloxacin correlations were moderate (0.40 < ρ < 0.7) only in Taiwan, the Philippines, and Turkey. Aminoglycosides-fluoroquinolones were moderately correlated (0.40 < ρ < 0.7) in South Africa, Thailand and Turkey. Linezolid was moderately correlated (0.40 < ρ < 0.7) with moxifloxacin in Turkey and ofloxacin in the Philippines.

WHO TB-priority groupings showed no discernible relationship with MIC correlation patterns (Supplementary Figures S2, S4, S5), as evidenced by the distinct drug-susceptibility profiles observed in countries with similar burden classifications, such as South Africa, India, and the Philippines (Supplementary Figure S5).

Comparing the percentage of DREAM isolates classified as resistant to each antibiotic (Supplementary Tables S4, S6) with these correlations suggests limitations of binary resistant-susceptible classifications in capturing the full complexity of resistance patterns within countries. While fluoroquinolones consistently showed strong correlations and similar resistance levels within countries, rifampicin and isoniazid displayed no correlation despite often similar resistance percentages in the DREAM database. A particularly noteworthy example is the case of bedaquiline-clofazimine in Thailand, where identical resistance percentages are contrasted with minimal correlation in MIC distributions.

### Characterizing bacterial MIC differences

**Figure 3:**
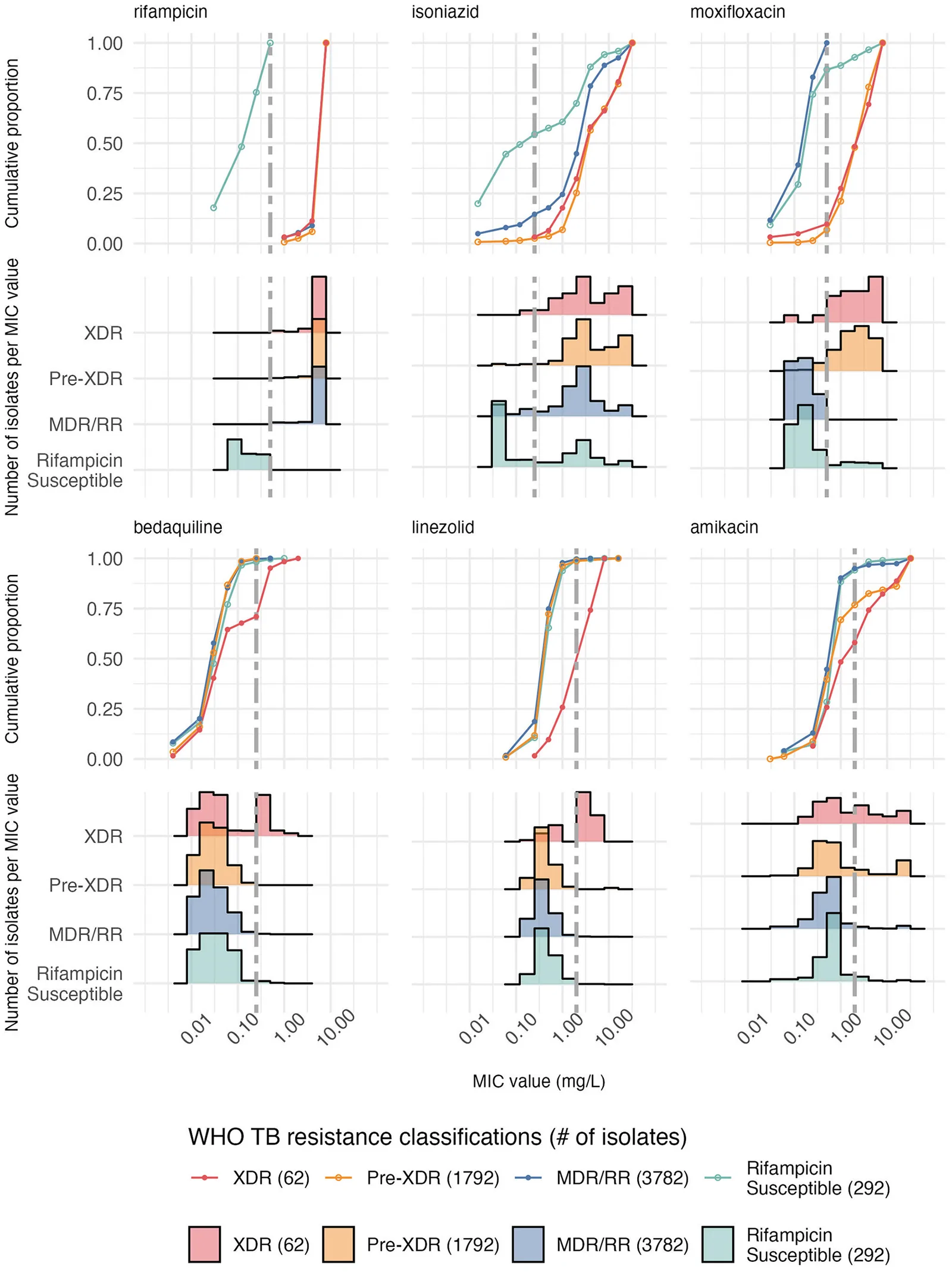
Cumulative proportion of isolates and histograms per MIC value across six antibiotics stratified by the 2024 WHO TB resistance classifications show an increase in MIC with resistance acquisition across all countries in the DREAM database (Supplementary Table S2). MDR/RR TB is rifampicin resistance with additional isoniazid resistance if tested for (here, all isolates with a rifampicin MIC above the threshold but not pre-XDR or XDR were included). Pre-XDR TB is MDR-TB + fluoroquinolone resistance (levofloxacin & moxifloxacin), and XDR-TB is Pre-XDR TB with additional resistance to bedaquiline or linezolid. “Rifampicin Susceptible” refers to an isolate with a rifampicin MIC lower than the cutoff present in the database. The antibiotics are ordered by resistance classifications, with the MDR (rifampicin + isoniazid) appearing first, followed by the pre-XDR (moxifloxacin) and then XDR (bedaquiline + linezolid). Amikacin is also included as resistance to it and other aminoglycosides, defined XDR-TB pre-2022 instead of bedaquiline and linezolid, including in Kaniga et al. The vertical gray dashed line is the epidemiological cut-off for each antibiotic, except for ethambutol, which has no reported epidemiological cut-off. Results for other antibiotics are shown in Supplementary Figure S6.

Our analysis reveals several deviations from expected stepwise resistance patterns, and the database held many “rifampicin-susceptible” isolates that were isoniazid-resistant and did not meet the MDR/RR definition (Figure 3). The distribution of rifampicin isolates by MIC value was as follows: 52 isolates at 0.03 mg/L, 89 isolates at 0.12 mg/L, 79 isolates at 0.25 mg/L, and 72 isolates at 0.5 mg/L. Most notably, we observed increasing MICs for isoniazid (IQR for MDR 2.0 mg/L vs. XDR 14.0 mg/L, Supplementary Table S5) visible as two peaks representing high and low resistance, as resistance classifications progress from MDR through XDR, despite the expectation that isoniazid resistance MICs should remain constant across MDR, pre-XDR and XDR definitions. Similar unexpected progressive increases in resistance are observed for amikacin (IQR for MDR 0.5 mg/L vs. XDR 6.4 mg/L), capreomycin (IQR for MDR 1.0 mg/L vs. XDR 6.0 mg/L), kanamycin (IQR for MDR 2.0 mg/L vs. XDR 6.0 mg/L) and clofazimine (IQR for MDR 0.11 mg/L vs. XDR 0.44 mg/L) (Figure 3, Supplementary Figure S6, Supplementary Table S5). In contrast, moxifloxacin (IQR for MDR 0.13 mg/L vs. XDR 7.0 mg/L), bedaquiline (IQR for MDR 0.03 mg/L vs. XDR 0.47 mg/L), linezolid (IQR for MDR 0.5 mg/L vs. XDR 5.25 mg/L), levofloxacin (IQR for MDR 0.25 mg/L vs. XDR 4.0 mg/L and ofloxacin (IQR for MDR 0.5 mg/L vs. XDR 8.0 mg/L) demonstrate patterns that align with definition expectations (Figure 3, Supplementary Figure S6, Supplementary Table S5). However, moxifloxacin similarly displays an unexpected secondary peak not observed for levofloxacin or ofloxacin (Figure 3, Supplementary Figure S6) despite theoretical expectations of equivalent resistance profiles between pre-XDR and XDR isolates (Figure 3, Supplementary Figure S6). During the study period, capreomycin, kanamycin, and amikacin could have been administered as a treatment for MDR/XDR-TB.

### Bedaquiline treatment implications

**Figure 4:**
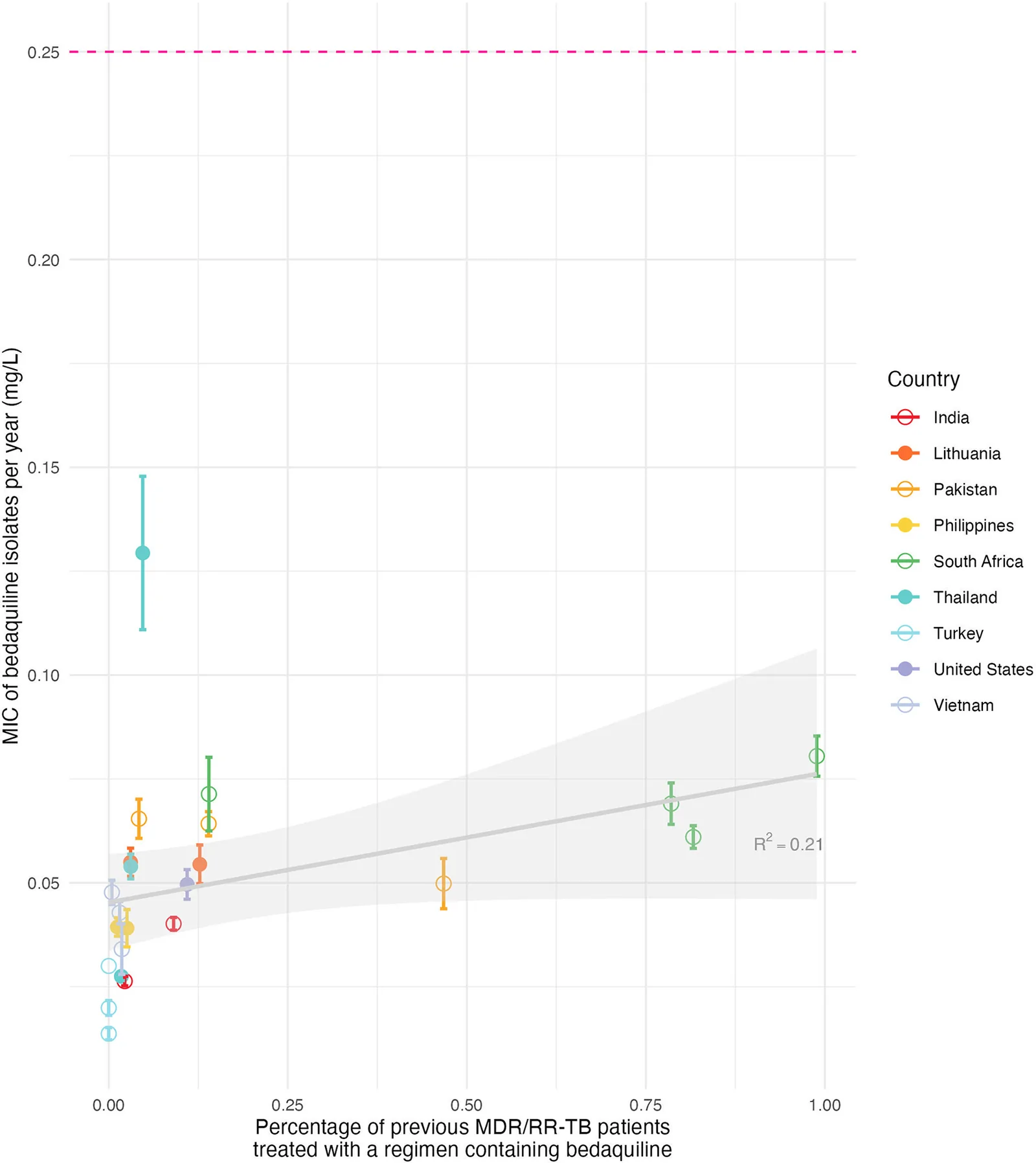
The increase in bedaquiline use shows little relation to the level of bedaquiline resistance as measured by country-level mean bedaquiline MIC. The percentage of MDR/XDR-TB patients who started on treatment containing bedaquiline in that country in the same year as the isolate was sampled, sourced from the WHO (x-axis), is shown against the MIC distribution for bedaquiline from isolates sampled in that country in the same year (y-axis). Each colored dot is the mean MIC per country, with bars indicating the standard error for each mean MIC value. The gray line is a fitted linear regression line, with the shaded area as the 95% confidence interval for the mean susceptible MIC per year vs. the percentage of bedaquiline use, with an R^2^ value of 0.21. The pink dotted line at the top represents the epidemiological cut-off for resistance. Results per country are shown in Supplementary Figure S7.

The mean MIC for bedaquiline isolates across the nine countries with data on bedaquiline use ranged from 0.01 to 0.13 mg/mL (Figure 4). As the fitted regression line indicates, bedaquiline resistance correlates weakly with the amount of bedaquiline administered between 2015 and 2019, accounting for only a small portion of the observed variation in the MICs (*R*^2^ value of 0.21). There was not enough bedaquiline use data from the WHO to perform this analysis on a country-level basis, which is evident when each country is viewed independently (Supplementary Figure S7).

## Discussion

Our study assessed the variation in drug-susceptibility, measured by MIC distributions for twelve antibiotics from *M. tuberculosis* isolates isolated from bedaquiline-treatment naive individuals across 11 countries. Significant between-country variation was observed in the distributions, with fluoroquinolones and isoniazid showing the greatest heterogeneity. Correlations between antibiotics per country revealed that six countries had moderate positive bedaquiline-clofazimine correlations, alongside expected positive correlations within antibiotic families. Analyzing MIC distributions by resistance class showed unexpected MIC distribution changes, particularly for XDR isolates with higher-than-expected MICs for isoniazid, amikacin, capreomycin, kanamycin and clofazimine. We found a weak relationship between historic bedaquiline use and bedaquiline MICs. Our findings reveal the substantial variation in MICs of *M. tuberculosis*, with distinct patterns emerging across different countries, revealing extra insights beyond standard resistance classification.

Our finding that there is substantial variation in MIC distributions by country to most anti-TB drugs is likely driven by country-specific treatment policies and the factors which drive these policies. Whilst there are global guidelines from the WHO, subtleties in implementation can lead to differences in antibiotic use, and both societal and healthcare infrastructure will lead to differences in transmission. For example, an examination of TB policies in India, Pakistan, the Philippines, South Africa, Thailand, and Vietnam by the Stop TB Partnership and Médecins Sans Frontières in 2020 revealed adherence to WHO directives but differing levels of efficacy (36). Although WHO recommends bedaquiline as a component of standard treatment for MDR-TB as part of a new regimen of antibiotics (BPaL/M), implementation of these regimens varies significantly across countries due to limited access to bedaquiline, specific bedaquiline drug-susceptibility testing and dissimilar healthcare infrastructures (17). Our results suggest that these practical constraints in adopting global guidelines manifest in divergent MIC distributions, which a country can then use to understand where the context-specific threats from resistance may be.

A Spearman rank correlation analysis supports this need for context-specific understanding, revealing country-specific patterns in MIC relationships, with a notable positive moderate correlation between bedaquiline and clofazimine MICs across six countries, including South Africa, Taiwan, Pakistan, South Korea, Turkey and the US. Since bedaquiline's 2014 approval for MDR-TB treatment, cross-resistance between these drugs has been linked to the *Rv0678* gene mutations observed in patients who are naïve to bedaquiline or clofazimine, as in the DREAM study (37–41). Clinical observations also support that cross-resistance to clofazimine can emerge in patients exposed only to bedaquiline (42, 43), including in isolates not harboring the Rv0678 mutation (44). However, multiple additional mutations can confer elevated MICs to both antibiotics, and the absence of a correlation in certain countries may reflect limited circulation of strains harboring the *Rv0678* mutation (45). Given the currently low resistance prevalence, continued phenotypic testing of bedaquiline remains crucial, with phenotypic testing of clofazimine supporting bedaquiline resistance determination depending on the setting. The aminoglycoside MICs showed moderate positive correlations with both clofazimine and fluoroquinolones MICs in six countries, including Pakistan, the Philippines, Taiwan, Thailand, Turkey and Vietnam, with Turkey demonstrating an additional moderate correlation between aminoglycoside and bedaquiline MICs. This pattern mirrors historical XDR-TB resistance definitions and indicates how resistance to clofazimine developed due to its use alongside aminoglycosides in previous XDR-TB treatments (46). These correlations, only detectable through MIC analysis rather than simple resistance classifications, potentially provide early warnings for newer regimens like BPaL/M, encouraging country-specific drug-susceptibility monitoring to optimize local treatment strategies.

The analytical method in this study is adapted from previous research that found different patient groups had substantial differences in MIC distributions for non-tuberculosis bacteria (20). The first analysis of the DREAM dataset used the MIC distributions to suggest modifications to the epidemiological cut-off for four antibiotics (23). Adapting the analytical method allowed us to build on the original DREAM study by analyzing all MIC distributions by country, including isolates not classed as MDR, and performing a Spearman rank correlation. By doing so, we have shown how MIC distributions differ by country and resistance class.

Our resistance class analysis indicated elevated MIC levels in pre-XDR and XDR isolates for antibiotics where a MIC increase may not be expected to occur, such as isoniazid, amikacin, capreomycin, kanamycin and clofazimine. These findings suggest that through treatment exposure, the acquisition of resistance to additional drugs may be accompanied by enhanced resistance to other drugs and drugs that define earlier resistance categories, rather than operating as independent events. Other authors have noted a rise in drug-resistance acquisition in *M. tuberculosis* strains already resistant to at least one antibiotic (47–49). In the case of isoniazid, we observed two distinct MIC peaks for high- and low-level resistance (50). Since MDR patients should not be exposed to isoniazid selective pressure during treatment, it is unclear what is driving the selection of high-isoniazid resistance. However, isolates from 2019 showed an increased proportion of low-level isoniazid resistance compared toprevious years. Although classifications such as “high-” or “low-level resistance” are rarely applied to other antibiotics, this may be useful, as similar patterns of “two-peaked” MIC distributions among resistant isolates are observed for drugs such as amikacin. The elevated amikacin MICs seen here are concerning as it remains the only recommended aminoglycoside alongside streptomycin after the WHO removed kanamycin and capreomycin from treatment guidelines in 2018 due to their association with treatment failure and relapse (51–53). Misdiagnosing TB resistance may lead to inappropriate antibiotic exposure, which may select highly resistant strains that confer resistance to multiple antibiotics.

MIC distributions at the country level are particularly informative, as they provide insight into the phenotypes of transmitting strains and enable correlation analyses that can guide tailored diagnostic strategies. For example, if moxifloxacin and amikacin MICs are correlated in a country with prevalent amikacin resistance, standard DST should include moxifloxacin, enabling early resistance detection and reducing the risk of treatment failure. Because these correlations differ between countries, country-level recommendations should include specific DST policies following a country's own drug susceptibly profiles. As in South Africa, consistently higher MICs indicate that controlling transmission of drug-resistant TB should be the primary focus, whereas countries with elevated MICs but less widespread resistance, such as Lithuania or Taiwan, should prioritize monitoring drug susceptibility to track emerging resistance. Current guidelines also focus on rifampicin susceptibility testing, yet many rifampicin-susceptible isolates in the DREAM database were resistant to other drugs. Therefore, TB DST guidelines should be revised at a local level to address resistance which may be going undetected.

Bedaquiline MIC distributions showed substantial country-level heterogeneity despite all isolates originating from patients who had not previously been treated with bedaquiline. This suggests that strains with mutations conveying an increased survival in the presence of bedaquiline may be being transmitted. On one hand, past use during clinical trials exposed more patients to bedaquiline in South Africa than in other countries, and MICs for bedaquiline were often highest in South Africa among the bedaquiline-naive patients in the DREAM dataset. This suggests that transmission of bedaquiline-resistant strains may be occurring, a finding corroborated by other researchers (13). On the other hand, our analysis relating bedaquiline-MICs to bedaquiline use across all countries in the dataset did not show strong evidence of a relationship. This may reflect that changes in bedaquiline MICs are driven more by the transmission of specific *M. tuberculosis* strains than by direct selective pressure from bedaquiline exposure. Alternatively, diagnostic limitations or country-differences in bedaquiline use may explain the weakness of this correlation. Nonetheless, this finding suggests we may yet observe a widespread increase in MICs as BPaL/M rollout progresses. Monitoring bedaquiline MIC distributions may be valuable in detecting MIC “creep” and serve as an early warning for developing bedaquiline resistance, whether due to transmission or *de novo* mutation.

We used the EUCAST-defined ECOFF of 0.25 mg/L for bedaquiline to classify isolate susceptibility. However, this threshold has been questioned, as numerous mutations have been associated with slight MIC increases that may fall below this cutoff yet are linked to poor treatment outcomes (54, 55). As a result, the current ECOFF may underestimate the prevalence of resistance. Several studies have proposed a lower ECOFF of 0.125 mg/L to more accurately define the wild-type distribution and improve resistance detection (23, 39, 56). Whilst this lower ECOFF may highlight more bedaquiline resistance, it does not address the genetic heterogeneity related to poor treatment outcomes. Therefore, phenotypic DST for bedaquiline should be continued alongside genetic sequencing to correlate specific mutations with changes in MIC values and to develop a clearer understanding of what constitutes resistance to bedaquiline. Where this is not possible, or only phenotypic DST is available, a Spearman rank correlation as presented here could be employed to estimate bedaquiline susceptibility.

Previous research on MIC distributions in TB has primarily focused on comparing phenotypic and genotypic resistance patterns, evaluating epidemiological cutoffs, and analyzing within-country drug-susceptibility (56–67). While large-scale studies such as the data compendium by the CRyPTIC consortium have collected MIC data across multiple countries, they have not specifically examined country-level differences in MIC distributions (67). This gap exists mainly because comparing MIC distributions between countries requires standardized laboratory protocols, only available in major initiatives like DREAM and CRyPTIC. Our analysis demonstrates the value of such cross-country comparisons, and with both studies making their data publicly available, similar analyses can be conducted in the future to understand geographical variations in drug-resistance patterns.

The DREAM database has several key limitations. First, it lacks linked patient data (age, sex, treatment history) and genomic information (lineage, resistance mutations). Second, there is potential for selection bias as the selection criteria for countries and isolates beyond being bedaquiline-naïve MDR/RR-TB patients are unclear, making it difficult to assess the dataset's representativeness. It is not clear if other patient characteristics such as age, sex, disease progression, HIV status or treatment history were representative of TB patients in each country or were comparable across countries. We make conclusions on MIC distribution variability at a country level, but do not know whether this truly reflects a country's MIC distribution. Moreover, the DREAM dataset included only isolates shown to be rifampicin-resistant from an initial screen, though 292 isolates were subsequently shown to have MICs below the cutoff. The 12,383 MICs reported with qualifiers limit our ability to precisely characterize variation, particularly at high concentrations such as for rifampicin. Assigning these values to the next-highest or lowest reported level may slightly under- or overestimate MICs; however, because MICs are constrained by the laboratory testing concentrations, such adjustments are unlikely to substantially affect interpretation except when many isolates cluster at the distribution extremes. While the study includes countries with varying TB burdens across different geographical regions and income groups, we cannot make firm country-level conclusions due to potential selection bias. Additionally, while focusing on bedaquiline-naïve patients allows us to study baseline MIC distributions without selective pressure, it excludes patients with prior bedaquiline exposure. Some degree of MIC variation below the ECOFF may be attributable to experimental error. However, given the standardized protocols and quality-controlled procedures employed in the DREAM study, such variation is more plausibly indicative of underlying biological differences. Nonetheless, a shift of one doubling dilution remains within the expected experimental margin of error. This could account for the observation that 72 isolates with a rifampicin MIC of 0.5 mg/L (the ECOFF threshold) were classified as susceptible, despite the possibility that some may harbor undetected resistance.

Future research within countries could examine how MICs change over time and in different sub-national settings to identify settings at risk of increased MICs, resistance, exposure or patient groups (bedaquiline-naive, women, etc.) (20). Future analysis could explore the contribution of *M. tuberculosis* lineages to MIC changes, as certain lineages are associated with increased transmissibility and resistance acquistion (68, 69). Our method may be most beneficial for countries exploring how, where and in whom resistance increases or transmits. The link between antibiotic usage and MIC levels could be further expanded with patient treatment history, which may be achievable in a more local setting.

Our research shows clear variations in MIC distributions across countries and resistance classes, especially for fluoroquinolone antibiotics. It provides evidence of distinct resistance dynamics per country, with evidence of bedaquiline-clofazimine MIC correlations in several countries and unexpected high-value MIC distributions in XDR-TB for antibiotics outside the current XDR definition. This expands the evidence on country differences in DR-TB and supports further targeted policy development, such as country-specific drug-susceptibility monitoring, to optimize local treatment strategies. Our findings highlight the need for tailored national strategies to combat DR-TB, emphasizing that global policies must be supplemented with targeted interventions for effective TB control and eradication.

## Data Availability

The data analyzed in this study is subject to the following licenses/restrictions: To obtain Johnson & Johnson Family of Companies' AMR surveillance data for analysis, researchers should use the AMR Register platform to submit a request. The anonymized data will then be made available to the researcher to download. Requests to access these datasets should be directed to amr@vivli.org or visit https://amr.vivli.org.
