# Supplementary material for: Country-level heterogeneity in MDR-TB drug susceptibility supports country-specific policy development
Source: Front Tuberc. 2025 Sep 25;3:1667354. doi: 10.3389/ftubr.2025.1667354 (PMC13491440; doi:10.3389/ftubr.2025.1667354)
Supplement: Supplementary file 1 [file Data_Sheet_1.pdf]

Figure S1 3
Figure S2 3
Figure S3 4
Figure S4 5
Figure S5 6
Figure S6 8
Figure S7 8

Table S1 10
Table S2 10
Table S3 10
Table S4 11

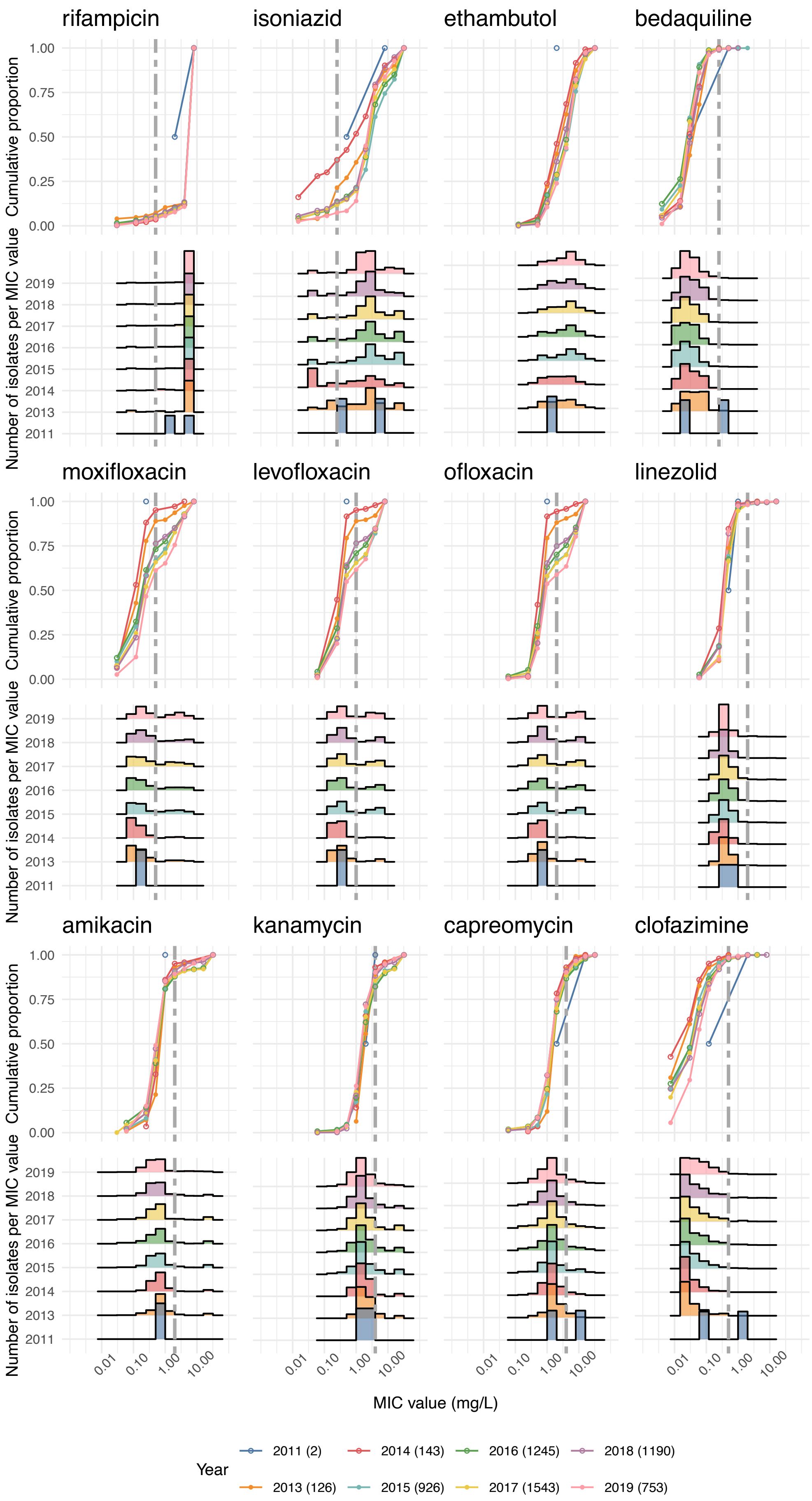
rifampicin
isoniazid
ethambutol
bedaquiline
moxifloxacin
levofloxacin
ofloxacin
linezolid
amikacin
kanamycin
capreomycin
clofazimine
Cumulative proportion
Number of isolates per MIC value
0.00
0.25
0.50
0.75
1.00
2011
2013
2014
2015
2016
2017
2018
2019
0.01
0.10
1.00
10.00
MIC value (mg/L)
Year
2011 (2)
2013 (126)
2014 (143)
2015 (926)
2016 (1245)
2017 (1543)
2018 (1190)
2019 (753)

Figure S1 The cumulative proportion of isolates per MIC value stratified by year is shown for each year, paired with histograms of the MIC values per year. The fluroquinolones appear to have an increase in MICs across time, whilst resistant isoniazid isolate MICs reduce. The vertical dashed line is the epidemiological cut-off for each antibiotic, except for ethambutol, which has no reported epidemiological cut-off (Table S1).

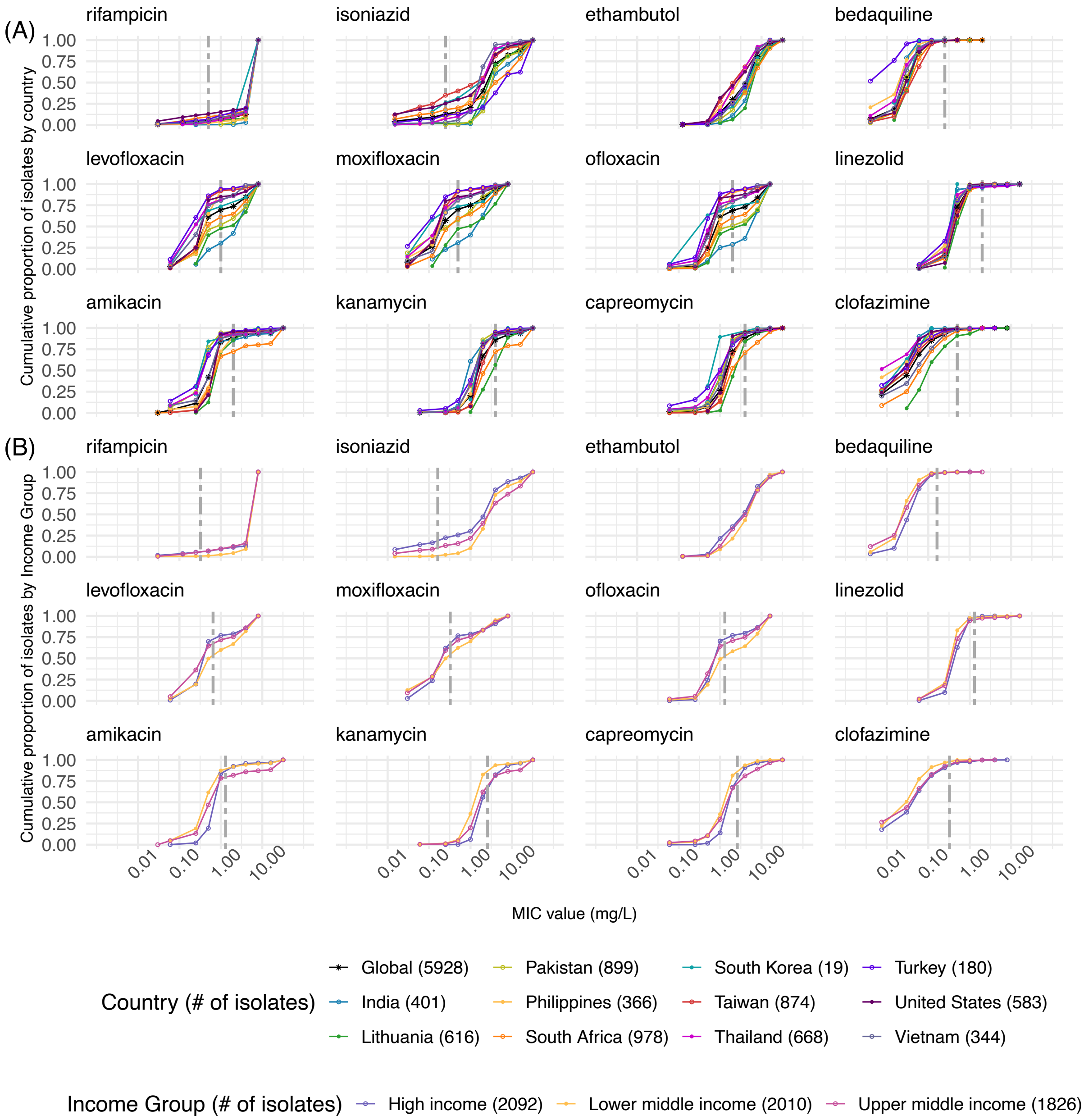

Figure S2 (A) Cumulative proportion of isolates per MIC value stratified by countries shows that each country has a distinctive antibiotic susceptibility profile. The vertical dashed line is the epidemiological cut-off for each antibiotic, except for ethambutol, which has no reported epidemiological cut-off (Table S1). The antibiotics are ordered by a family with the aminoglycosides (Amikacin, Capreomycin, Kanamycin) appearing first, followed by the fluoroquinolones (levofloxacin, moxifloxacin, ofloxacin). The legend includes the number of

isolates per country in the dataset. (B) The cumulative proportion of isolates per MIC value stratified by income group in 2025 shows little distinction between groups.

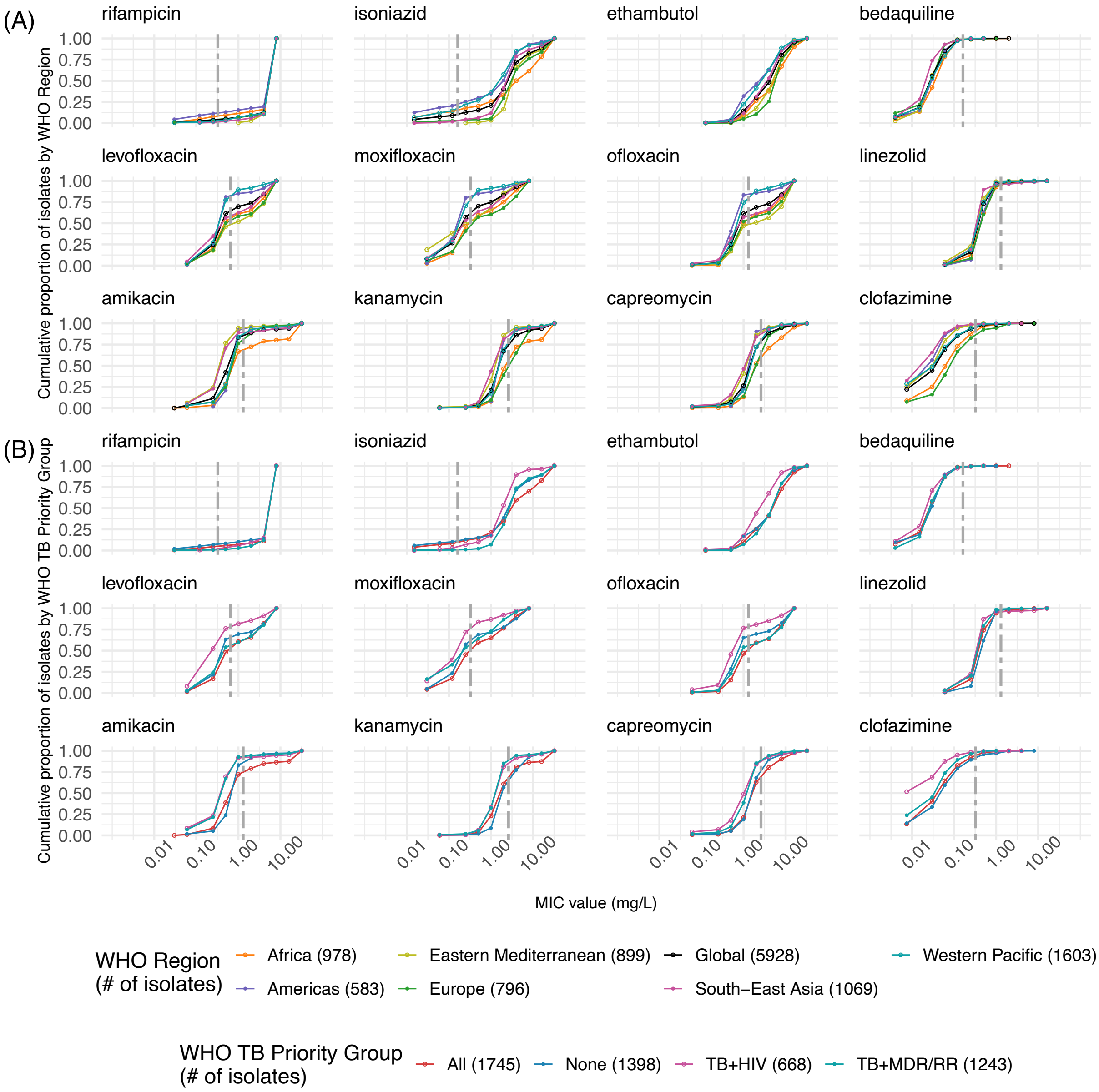

Figure S3 (A) Cumulative proportion of isolates per MIC value stratified by WHO region shows each region has a distinctive antibiotic susceptibility profile for most antibiotics, more visible than the continent grouping. (B) The cumulative proportion of isolates per MIC value stratified by WHO TB priority group in 2021-2025 shows that each group has a distinctive antibiotic susceptibility profile, which aligns with the priority group's intentions. The vertical dashed line is the epidemiological cut-off for each antibiotic, except for ethambutol, which has no reported epidemiological cut-off (Table S1). Note: TB+HIV only includes Thailand.

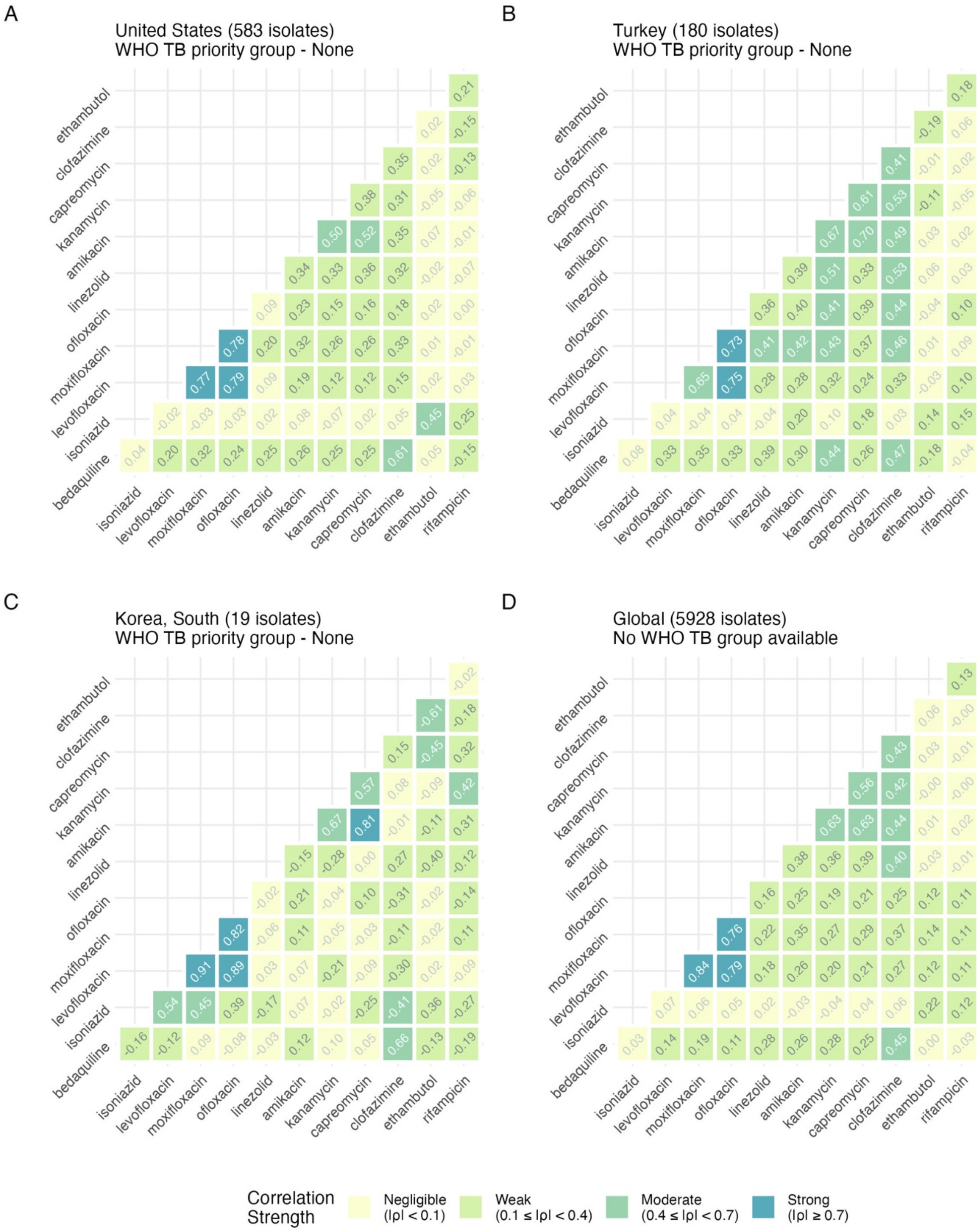

Figure S4 Spearman rank correlation analysis of antibiotic pairs within isolates by country reveals distinct relationships. Each panel (A-D) represents a country, with each tile containing the correlation result between each antibiotic shown on the axes. A higher value and a darker colour represent a higher correlation. All correlations are coloured along the same scale for easy comparison. The number of isolates per country is shown in the title, and where it exists, the WHO TB priority group is also indicated.

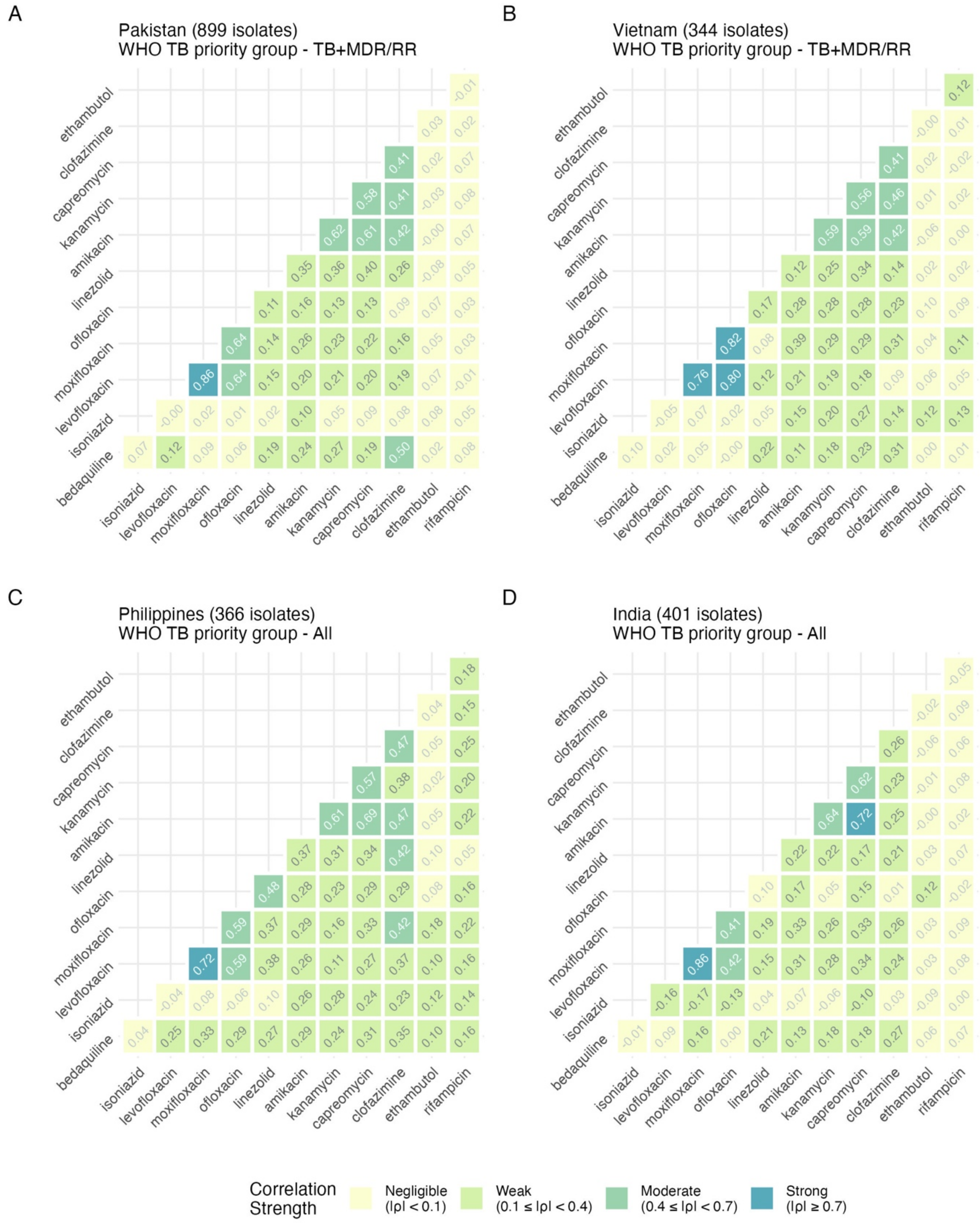

Figure S5 Spearman rank correlation analysis of antibiotic pairs within isolates by country reveals distinct relationships. Each panel (A-D) represents a country, with each tile containing the correlation result between each antibiotic shown on the axes. A higher value and a darker colour represent a higher correlation. All correlations are coloured along the same scale for easy comparison. The number of isolates per country is shown in the title, and where it exists, the WHO TB priority group is also indicated.

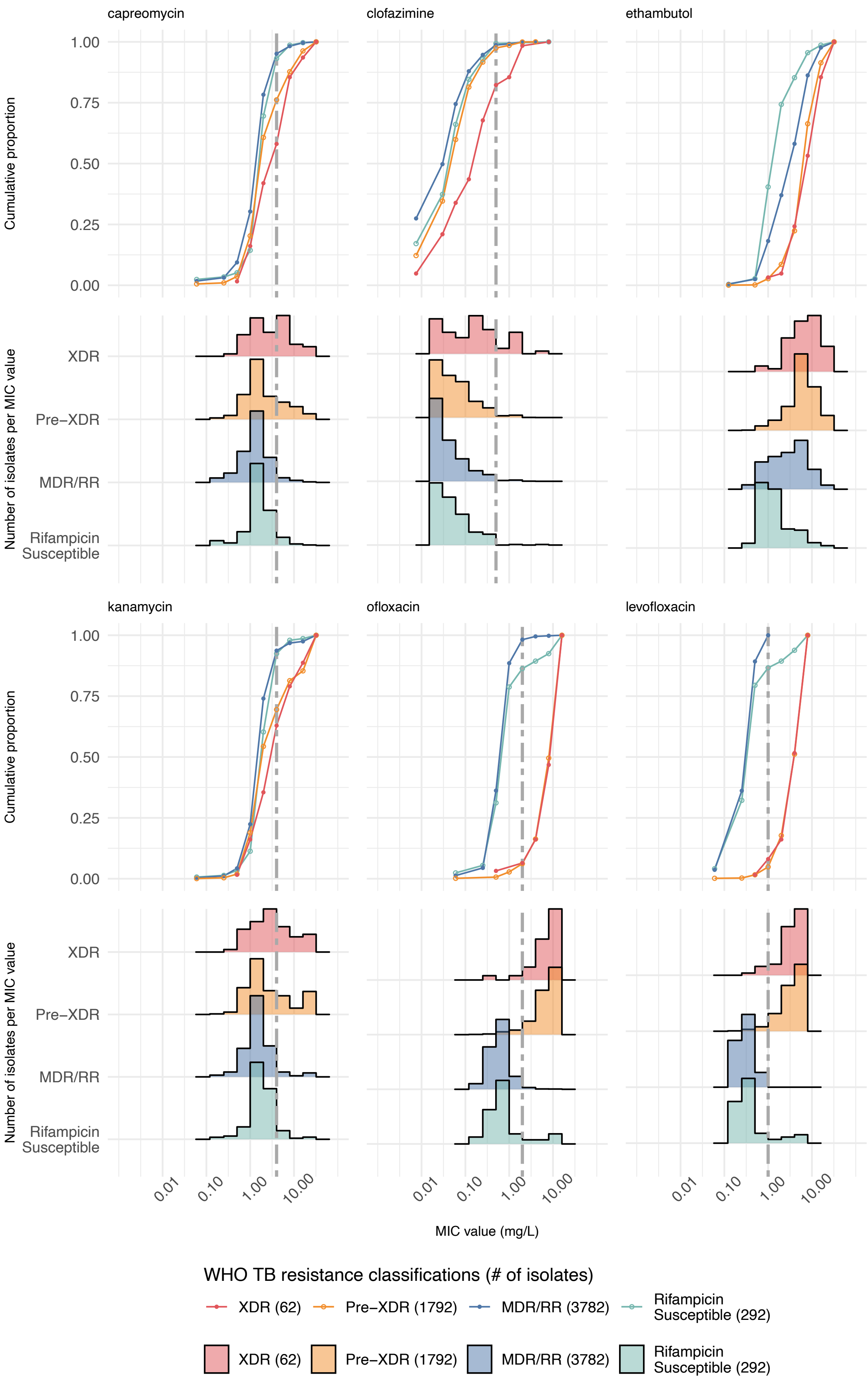
capreomycin
clofazimine
ethambutol
kanamycin
ofloxacin
levofloxacin
Cumulative proportion
1.00
0.75
0.50
0.25
0.00
Number of isolates per MIC value
XDR
Pre−XDR
MDR/RR
Rifampicin Susceptible
0.01
0.10
1.00
10.00
MIC value (mg/L)
WHO TB resistance classifications (# of isolates)
XDR (62)
Pre−XDR (1792)
MDR/RR (3782)
Rifampicin Susceptible (292)

Figure S6 Additional antibiotics not shown in Figure 3: Cumulative proportion of isolates per MIC value paired with kernel density plots stratified by the 2024 WHO TB resistance classifications show an increase in MIC with resistance acquisition across all countries in the DREAM database (Table S1). The definitions of MDR, Pre-XDR and XDR are: MDR/RR TB is rifampicin resistance with additional isoniazid resistance if tested for (here all isolates with a rifampicin MIC above the threshold but not pre- or XDR were included), Pre-XDR TB is MDR-TB + fluoroquinolone resistance, and XDR-TB is MDR-TB with additional fluoroquinolone resistance and resistance to bedaquiline or linezolid. "Rifampicin Susceptible" refers to an isolate with a rifampicin MIC lower than the cutoff present in the database. The antibiotics are ordered by resistance classifications, with the MDR (rifampicin + isoniazid) appearing first, followed by the pre-XDR (moxifloxacin) and then XDR (bedaquiline + linezolid, as well as amikacin, as this was in the pre-2024 WHO XDR definition). The vertical dashed line is the epidemiological cut-off for each antibiotic, except for ethambutol, which has no reported epidemiological cut-off (Table S1).

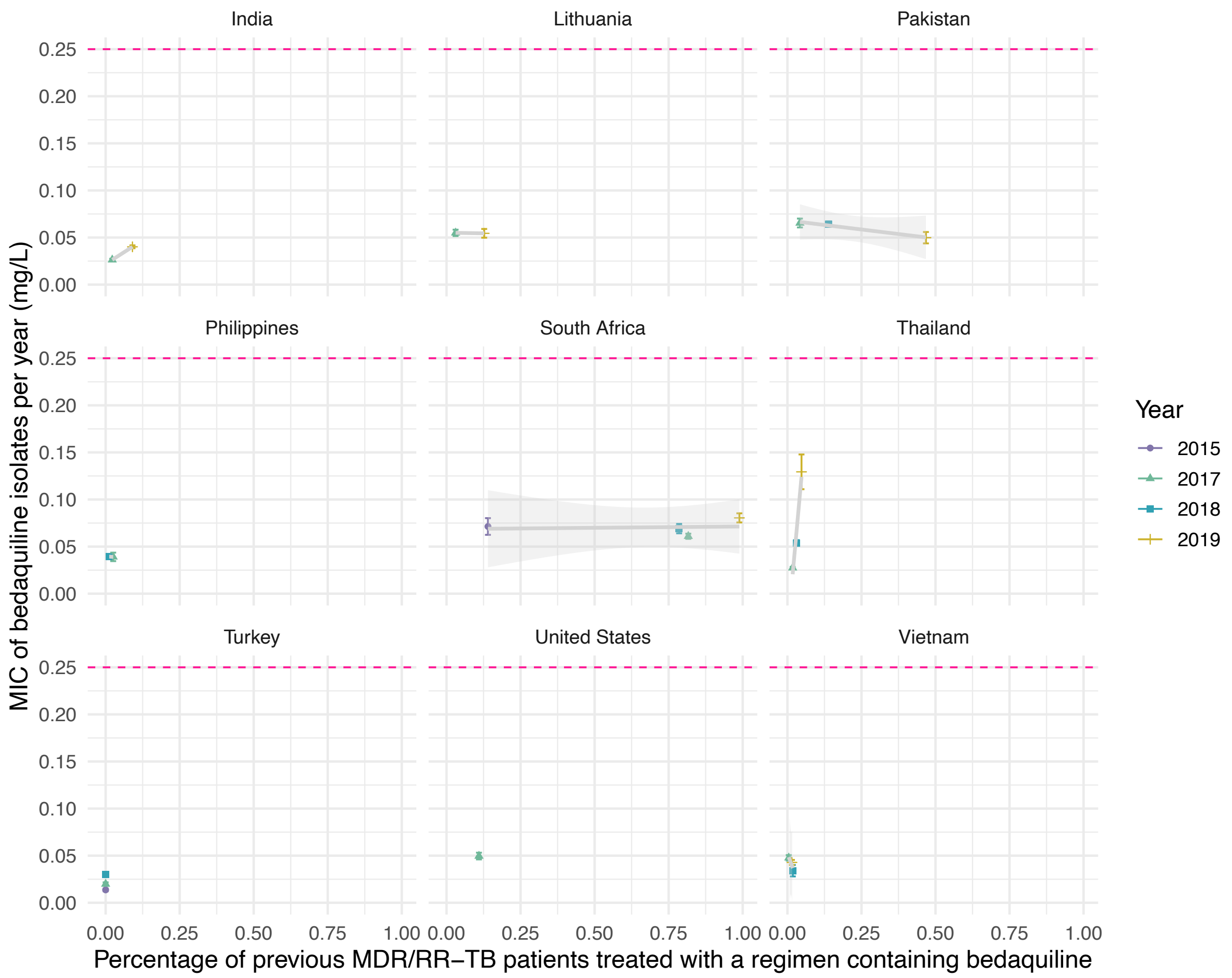

Figure S7 There was not enough bedaquiline use data from the WHO for a country-level analysis of bedaquiline use and the average bedaquiline MIC per country. The percentage of MDR/XDR-TB patients who have started on treatment containing bedaquiline in the same year as the isolate was sampled, sourced from the WHO (x-axis), is shown against the MIC distribution for bedaquiline from isolates sampled in that country in the same year (y-axis). Each coloured dot is the mean susceptible MIC per country per year, with bars indicating the standard error for each mean MIC value. The grey line is a fitted linear regression line,

with the shaded area as the standard error (95% confidence interval) for the mean susceptible MIC per year vs the percentage of bedaquiline use. The pink dotted line at the top represents the epidemiological cut-off for resistance.

Table S1 Epidemiological cut-off per antibiotic

Antibiotic	Epidemiological cut-off sourced from Kaniga et al. 2022 and EUCAST (mg/L)
bedaquiline	0.25
isoniazid	0.25
levofloxacin	1
moxifloxacin	0.5
ofloxacin	2
linezolid	2
amikacin	2
kanamycin	4
capreomycin	4
clofazimine	0.5
ethambutol	NA
rifampicin	0.5

Table S2 Additional groupings per country

Country	WHO Region	WHO TB Priority Group	Income Group
India	South-East Asia	All	Lower middle income
Lithuania	Europe	None	High income
Pakistan	Eastern Mediterranean	TB+MDR/RR	Lower middle income
Philippines	Western Pacific	All	Lower middle income
South Africa	Africa	All	Upper middle income
South Korea	Western Pacific	None	High income
Taiwan	Western Pacific	NA	High income
Thailand	South-East Asia	TB+HIV	Upper middle income
Turkey	Europe	None	Upper middle income
United States	Americas	None	High income
Vietnam	Western Pacific	TB+MDR/RR	Lower middle income
Global	Global	NA	Global

Table S3 Number of isolates per sub-group for analysed groupings in the dataset, excluding the Global category.

Grouping	Sub-group	Number of isolates
Resistance class	MDR/RR	3714
	Pre-XDR	1836
	XDR	86
	Rifampicin susceptible	292
WHO Region	Western Pacific	1603
	South-East Asia	1069
	Africa	978
	Eastern Mediterranean	899
	Europe	796
	Americas	583

Country	South Africa	978
	Pakistan	899
	Taiwan	874
	Thailand	668
	Lithuania	616
	United States	583
	India	401
	Philippines	366
	Vietnam	344
	Turkey	180
	Korea, South	19
WHO TB Priority	All	1745
	None	1398
	TB+MDR/RR	1243
	TB+HIV	668
Income Group	High income	2092
	Lower middle income	2010
	Upper middle income	1826
Year	2011	2
	2013	126
	2014	143
	2015	926
	2016	1245
	2017	1543
	2018	1190
	2019	753

Table S4 Percentage of resistant isolates across all countries (Global) or per country for all 12 antibiotics in the DREAM database. Resistance was determined by MICs equalling or exceeding the epidemiological cut-off. The value is the percentage of isolates resistant to the antibiotic in this country.

Antibiotic	Global	Lithuania	Pakistan	South Africa	Taiwan	Thailand
bedaquiline	0.56	0.97	0.89	0.92	0.34	0.75
isoniazid	87.2	98.38	99.67	82.11	64.99	92.96
levofloxacin	30.38	52.27	48.05	38.85	7.78	18.26
moxifloxacin	29.76	52.76	42.27	40.49	8.12	16.32
ofloxacin	14.84	20.94	18.8	19.02	2.97	10.63
linezolid	1	0.65	0.22	1.64	0.34	1.05
amikacin	10.26	13.96	4	26.28	4.92	6.89
kanamycin	6.01	32.14	0.67	6.95	2.63	2.54
capreomycin	6.04	9.74	3	12.17	4.58	2.99
clofazimine	1.82	9.25	0	3.27	0.69	0.75
rifampicin	95.07	95.29	100	90.49	96	96.56

Antibiotic	United States	India	South Korea	Philippines	Turkey	Vietnam
bedaquiline	0.34	0	0	0	0	0
isoniazid	74.61	100	73.68	90.98	88.33	96.8
levofloxacin	14.92	69.58	26.32	7.92	6.11	19.19
moxifloxacin	15.09	69.08	26.32	9.29	8.33	18.6
ofloxacin	6.86	39.4	5.26	6.01	6.11	19.48
linezolid	0	3.99	0	1.64	1.11	0.87
amikacin	4.12	13.47	15.79	9.29	2.78	5.81
kanamycin	1.54	2.49	10.53	3.28	3.33	1.45
capreomycin	1.55	7.48	10.53	6.83	3.89	5.52
clofazimine	0	0	0	0.55	1.11	1.16
rifampicin	86.96	99.75	100	98.09	93.33	95.35
